# Cold denaturation induces inversion of dipole and spin transfer in chiral peptide monolayers

**DOI:** 10.1038/ncomms10744

**Published:** 2016-02-26

**Authors:** Meital Eckshtain-Levi, Eyal Capua, Sivan Refaely-Abramson, Soumyajit Sarkar, Yulian Gavrilov, Shinto P. Mathew, Yossi Paltiel, Yaakov Levy, Leeor Kronik, Ron Naaman

**Affiliations:** 1 Department of Chemical Physics, Weizmann Institute of Science, Rehovoth 76100, Israel; 2 Department of Materials and Interfaces, Weizmann Institute of Science, Rehovoth 76100, Israel; 3 Department of Structural Biology, Weizmann Institute of Science, Rehovoth 76100, Israel; 4 Department of Applied Physics and Center for Nano Science and Nanotechnology, The Hebrew University, Jerusalem 91904, Israel

## Abstract

Chirality-induced spin selectivity is a recently-discovered effect, which results in spin selectivity for electrons transmitted through chiral peptide monolayers. Here, we use this spin selectivity to probe the organization of self-assembled α-helix peptide monolayers and examine the relation between structural and spin transfer phenomena. We show that the α-helix structure of oligopeptides based on alanine and aminoisobutyric acid is transformed to a more linear one upon cooling. This process is similar to the known cold denaturation in peptides, but here the self-assembled monolayer plays the role of the solvent. The structural change results in a flip in the direction of the electrical dipole moment of the adsorbed molecules. The dipole flip is accompanied by a concomitant change in the spin that is preferred in electron transfer through the molecules, observed via a new solid-state hybrid organic–inorganic device that is based on the Hall effect, but operates with no external magnetic field or magnetic material.

## Abstract (web summary)

Spin selectivity for electron transport through peptide monolayers depends on the dipole moment of the system. Here, the authors show that self-assembled monolayers of peptides with α-helix structures transform to more linear structures upon cooling, inducing a flip in the direction of dipole moment and a change in preferred spin for electron transport.

## Graphical abstract

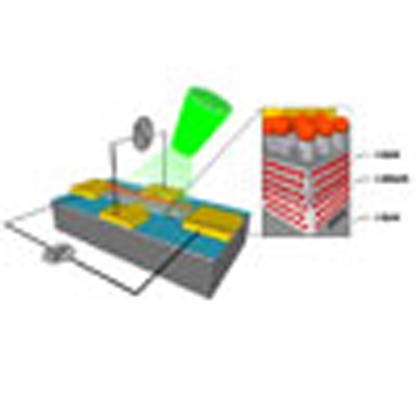

Self-assembled monolayers (SAM) have been studied intensively due to their technological importance in various fields, from organic electronics to friction and catalysis (see, for example, refs 1, 2, 3, and references therein). Commonly it is assumed that the structure of the molecules within the SAM is only weakly dependent on their supra-molecular assembly. However, this is not necessarily the case, especially when dealing with SAM based on molecules well-known to exhibit structural diversity, such as oligopeptides.

Experiments performed more than a decade ago showed that the spin selectivity of photoelectrons transmitted (from a gold substrate) through an oligopeptide monolayer is affected by the direction of the dipole moment of the molecules relative to the electrons' velocity^4^ and that both are temperature-dependent^5^. These results are summarized in Fig. 1 for a gold-adsorbed monolayer of oligoalanine consisting of 22 amino acid units. By monitoring the work function of the sample (by means of its contact potential difference (CPD), with a reference gold surface) as a function of temperature, a change in the sign of the CPD is observed (Fig. 1a). Although the null CPD signal does not necessarily coincide with the dipole flipping point, owing to the Pauli Push-back effect that changes the dipole of a monolayer-adsorbed surface even in the absence of a molecular dipole^6^,^7^. this change does reflect a change in surface dipole^8^. The dipole flip correlates with a change in the sign of the preferred spin of the transmitted photoelectrons (Fig. 1b,c).

While the data of Fig. 1 may hint at a structural effect, the origin of the dipole and spin flipping remained unknown when these results were published. Indeed, determining structural changes in a monolayer at different temperatures, and correlating them with dipole and spin-filtering properties, is not a simple task.

Here, we augment the traditional characterization tools with a thorough computational investigation using molecular dynamics (MD) and density functional theory (DFT), as well as with a new experimental setup—the magnet-less Hall device, based on the recently discovered chirality-induced spin selectivity (CISS) effect^9^,^10^,^11^,^12^. The combined computational and experimental results reveal that the oligopeptide structure is highly temperature-dependent. We find that the surrounding chemical environment in the SAM can induce ‘denaturation' upon cooling, much as in biological systems ‘cold denaturation' can take place due to protein interaction with the surrounding water^13^, but here the SAM plays the role of the solvent. This dramatic structural change results in a flip in the direction of the dipole moment of the adsorbed molecules, which, in turn, reverses the polarity of the preferred spin in electron transfer experiments. This phenomenon is of importance not only for the basic understanding of the structure of adsorbed molecules, but also opens new possibilities for controlling spin in organic spintronic devices^14^.

## Results

### Computational results

Our working hypothesis at the outset of this investigation was that the oligopeptide chains may be entropy-driven to fold with increasing temperature. For investigating this initial idea, classical MD simulations were performed on a dense monolayer of 10-mer oligoalanine peptides attached covalently to a surface using the GROMACS package (see Methods section for details). Because the MD force fields are commonly calibrated at room temperature, simulations at very low temperatures are not necessarily reliable as they cannot be expected to represent the system energetics accurately. This difficulty was circumvented by avoiding the need to study the peptide chain explicitly over a wide temperature range. Instead, we tested the effect of folding directly by starting from two distinct organizations of the peptides in the self-assembled monolayer, an α-helical and an extended conformation. Each system was simulated for several nanoseconds at 300 K and was analyzed by focusing on the conformational energetics and dynamics of the central polyalanine peptide in the simulated monolayer. As shown in Fig. 2a, the two configurations of the polyalanine monolayer exhibited different properties, with the monolayer of the helical peptides being more disordered and less packed. We further used the distribution of the inter-peptide angle (Fig. 2c) to estimate the contribution of this degree of freedom for the configurational entropy. Using the simple relation *S*=−*k p*ln*p*, where *p* is the probability of the angle, we found that at room temperature this degree of freedom contributes 0.33 kcal mol^−1^ for the helical 10-mer monolayer but only 0.005 kcal mol^−1^ for the linear 10-mer monolayer. Confirming our hypothesis, the stability of the α-helix form at room temperature, thus, is indeed due to its relatively high entropy, while the linear form is more stable at low temperature because of its lower enthalpy. For example, the total number of hydrogen bonds for the central peptide is much larger when the peptide is linear than helical, as shown in Fig. 2b. The detailed calculations suggest that polyalanine adsorbed as a self-assembled monolayer is going through ‘cold denaturation' upon cooling and is gradually transformed from the α-helix structure to an extended β-strand shape.

To examine the relation between the cold denaturation and electrical properties, we ‘extracted' the central peptide from MD runs with various degrees of stretching and examined the gas-phase dipole and electronic structure obtained from DFT calculations. Importantly, these calculations were performed using the optimally-tuned range-separated hybrid scheme (see Methods section)^15^,^16^. This approach was shown to provide accurate results for the outer-valence electronic structure of finite systems in general^17^,^18^ and in particular predict their ionization potential and electron affinity from the highest occupied molecular orbital (HOMO) and lowest unoccupied molecular orbital (LUMO), respectively^15^,^16^,^19^,^20^,^21^,^22^,^23^, where standard schemes typically fail^24^,^25^. This is of unique importance for highly polar systems^26^, including protein and peptide molecules^27^,^28^,^29^, where a strongly underestimated gap may lead, under a large internal electric field, to spurious gap closure and qualitative failure of the calculation.

**Figure 1:**
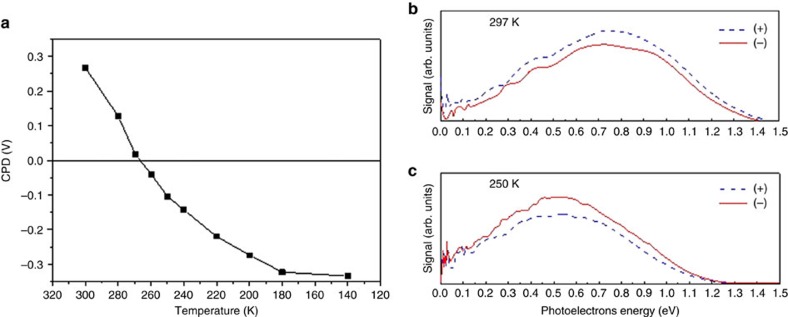
Temperature-dependent contact potential (surface work function) and spin-polarized photoemission. (**a**) Temperature- dependent contact potential difference (CPD) measurement of gold coated with a self-assembled monolayer of L-polyalanine, bound to the surface through the carbon terminal. Zero voltage indicates the CPD of the bare gold. (**b**,**c**) Energy distribution of photoelectrons ejected from the gold substrate using a 248 nm laser with clockwise (+, blue) or counter clockwise (−, red) circularly polarized light, at temperatures of 297 K (**b**) or 250 K (**c**). Adapted from ref. 5, used with permission.

**Figure 2:**
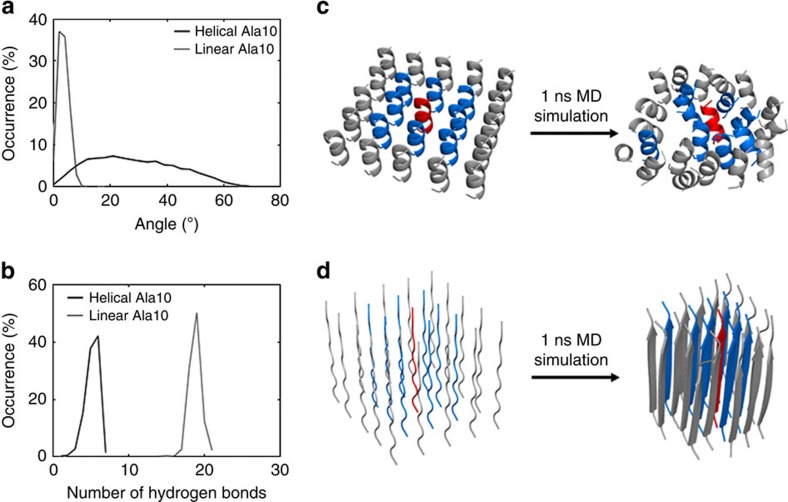
Molecular dynamics simulations of monolayers of oligoalanine peptides attached covalently to a surface. 5 × 5 monolayer of 10-mer oligoalanine (Ala10), modeled as (**c**) an α-helical or (**d**) a linear conformation. Each model of the monolayer was simulated for 1 ns and further analysis was performed on the central peptide (red) relative to its eight neighbors (blue). Also shown is the distribution of the: (**a**) angle of the central peptide intra-peptide hydrogen bonds; (**b**) number of inter-peptide hydrogen bonds between the central peptide and neighboring ones.

**Figure 3:**
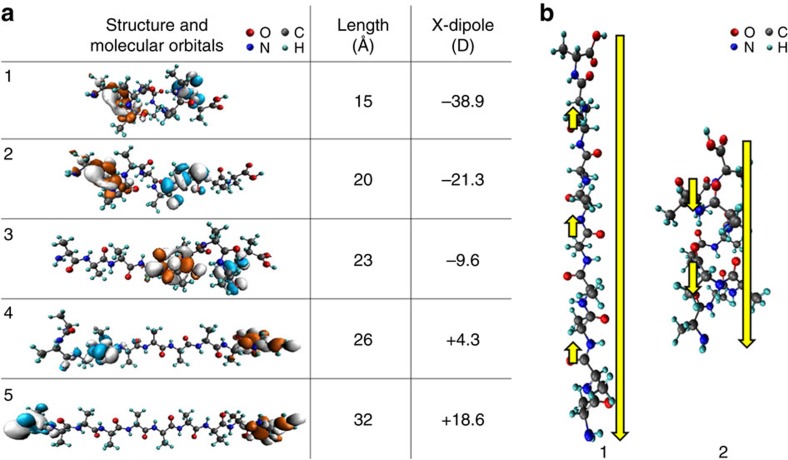
Calculated structures and dipole moments of oligoalanine molecules. (**a**) Several structures of 10-mer oligoalanine molecules, gradually stretched from their α-helix conformation to a linear one. For each structure, the molecular length (determined by the distance along the molecular axis between the two farthest C atoms), as well as the DFT-computed dipole moment along the molecular axis and the highest occupied (light blue) and lowest unoccupied (orange) molecular orbitals, are given. (**b**) The change in dipole direction upon structural conformation of 10-mer oligoalanines, demonstrated schematically for the most linear and most helical conformations of the examined peptides. Long arrows indicate the dipole formed between the C-terminus and the N-terminus of the peptide. Short arrows indicate local dipoles formed by peptide bonds.

Representative calculated structures of the model, 10-mer oligoalanine system are shown in Fig. 3a. Importantly, and in agreement with the early experiments of Fig. 1, we find that as the helical structure gradually unfolds and the terminal groups move further apart, the dipole becomes less negative and eventually changes sign. The flip in dipole direction upon structural change is not unique to this specific length of the peptide and also appeared in calculations of longer (20-mer) and shorter (4-mer) oligoalanines, not shown for brevity. The main effect causing the dipole flip is demonstrated schematically in Fig. 3b for a 10-mer oligoalanine. For a linear conformation, the individual contributions of the local dipole moments formed by each of the peptide bonds add up coherently along the molecular axis, in the direction opposite to the dipole formed between the C-terminus and the N-terminus of the peptide. In the α-helical form, the direction of the same peptide-bond related local dipoles is flipped, due to additional hydrogen bonds that set the directionality of the oxygen atoms and change the relative direction of the O–N pairs in the peptide bond. The contribution of these local dipoles to the net dipole is then in the same direction as the contribution of the C-terminus to N-terminus dipole. This results in a larger net dipole moment in the case of the α-helix structure, which has an opposite sign to that of the linear structure. Accordingly, two different structures of similar length can lead to different dipole moments with different signs, as demonstrated by structures 3 and 4 in Fig. 3a. Both structures are ∼25 Å long, but while in the somewhat shorter structure 3 many peptide bonds (which are closer to the C-terminus) are still within a helical form, in the somewhat longer structure 4 almost all peptide bonds are linearly aligned. This causes the dipole to flip sign along the molecular axis. Furthermore, the change of dipole sign is accompanied by a related flip in the position of the occupied and unoccupied orbitals, as visualized in Fig. 3a by the charge density distributions of the HOMO and LUMO. This can be easily rationalized in terms of quasi-electron and quasi-hole states responding oppositely to the electric field^30^.

### Experimental results

Encouraged by the above theoretical findings, we sought direct experimental evidence for chain elongation with decreasing temperature, through a combination of ellipsometry and FTIR (Fourier transform infrared spectroscopy see Methods section). Note that here and throughout this article, experiments were performed on {Ala-Aib} oligopeptides. These were chosen because they possess the same structure as the pure alanine-based ones, discussed so far, but exhibit better solubility. As shown below, they exhibit the exact same phenomena as the oligoalanine peptides. The IR spectroscopy showed consistently an increase in the absorption signal upon cooling to 14 K, with no significant changes observed below 100 K. However, while these data are consistent with the denaturation process, they do not constitute a clear indication for it. Ellipsometry data, given in Table 1, did provide clear evidence for chain elongation upon cooling. Experimentally, a length change that depends on the cooling time was observed. For very long cooling times the length is approximately doubled, in agreement with the theoretical prediction.

The remaining question, then, is the origin of the spin-transfer selectivity (Fig. 1b) and whether cold denaturation can also explain its polarity inversion. Generally, spin-selectivity in electron transmission or tunneling through chiral layers is explained by the CISS effect^9^,^10^,^31^, which has also been used to explain spin-specific electron transfer through oligopeptides^32^ and even suggested to play a role in electron transfer through biological systems^33^. Briefly, as an electron moves along a chiral molecule it experiences the electrostatic potential of the molecule, which is chiral. In the electron's rest frame, the charge that defines the electrostatic potential generates a magnetic field which splits the degeneracy of the electron spin states. The sign of the spin being transferred more efficiently through the chiral molecules depends on the sign of the electric field acting on the electron. This is because spin-selectivity is controlled by spin–orbit coupling, which has the well-known form, 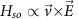, where 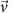 is the electron velocity and 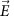 is the electric field. Because the dipole flip induced by the cold denaturation necessarily implies a change in sign of the overall electric field across the oligopeptide, reversal in spin-filtering polarity emerges naturally.

**Figure 4:**
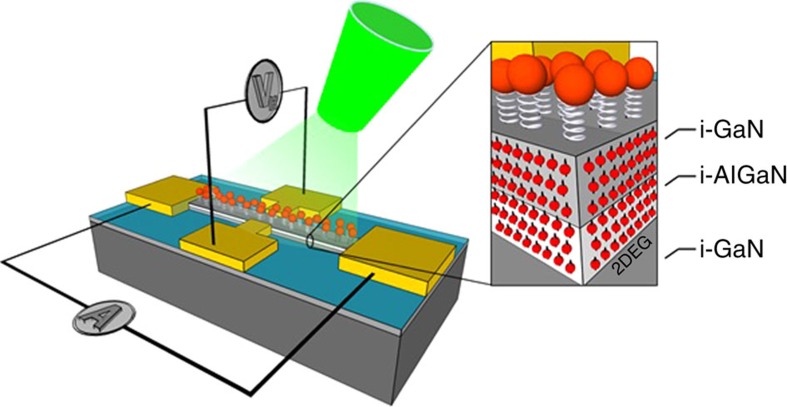
Schematic structure of a GaN-based magnetless Hall device. A two-dimensional electron gas is formed between the AlGaN layer and the GaN layer underneath it. Current flows between two electrodes and the Hall voltage is measured between the transverse set of electrodes. Nanoparticles are attached to the substrate through chiral molecules (in this case, oligopeptides) adsorbed on top of the conductive channel.

While the above explanation is plausible, proving experimentally that indeed the preferred spin inversion is inexorably linked to structural changes is more difficult. To pursue this, we developed a new type of Hall-effect device, shown in Fig. 4 (see Supplementary Fig. 1). The Hall effect is a long-known phenomenon^34^. Briefly, when current is flowing in a substrate between two electrodes, a magnetic field applied perpendicular to the current flow induces an electric potential perpendicular both to the current and the magnetic field. This effect is used as a standard tool for the study of properties of semiconductors, notably charge carrier concentration and mobility^35^. Here, we demonstrate that the spin-filtering capabilities of the chiral monolayer allow for the observation of the Hall effect without either an externally applied magnetic field or a permanent magnet. Instead, as explained below, the hall voltage arises from the accumulation of spin-transfer. This allows us to probe the relation between structural changes and spin inversion directly.

The device, shown schematically in Fig. 4, is based on a two-dimensional electron gas structure, onto which two sets of Hall probe electrodes were evaporated. We have chosen to implement the device on a GaN substrate due to the long spin-lifetime of this material^36^. The width and length of the conducting channel were 40 and 700 μm, respectively. On the conduction channel, a SAM of NH_2_-{Ala-Aib}_8_-COCHNH_2_CH_2_PO_4_H_2_ or SHCH_2_CH_2_CO-{Ala-Aib}_5_-COOH oligopeptides were adsorbed, that is, either a phosphonate or a carboxylate group was used to bind the oligopeptide to the GaN surface. The IR spectra of the adsorbed layer are shown in Supplementary Fig. 2. The oligopeptides chosen possess the same structure as the pure alanine-based ones discussed so far and were chosen because of their better solubility. CdSe nano particles (NPs) of ∼6 or ∼2 nm diameter were then attached to the amine or thiol tail. The SEM image of the layer with the nanoparticles is shown in Supplementary Fig. 3 and additional information is provided in the Supplementary Methods. A constant current of 10 μA was injected along the structure, with the Hall voltage measured in the transverse direction (see Methods section).

**Figure 5:**
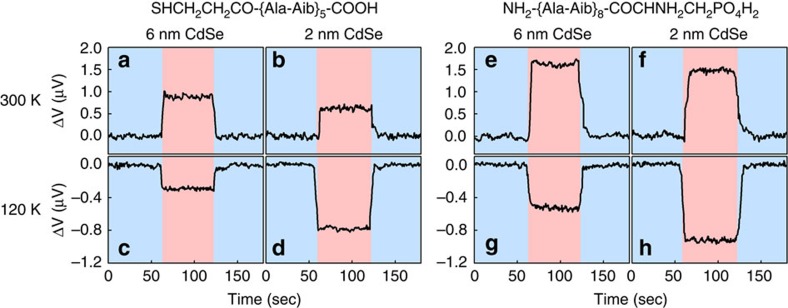
Hall voltage measurements at different temperatures and for different sizes of nanoparticles. All measurements were performed at 300 K and at 120 K for devices of the type shown in Fig. 4, coated with 6 and 2 nm diameter CdSe nanoparticles that are attached to the GaN surface via NH_2_-{Ala-Aib}_8_-COCHNH_2_CH_2_PO_4_H_2_ or SHCH_2_CH_2_CO-{Ala-Aib}_5_-COOH molecules, at a current of 10 μA. The devices were illuminated with a 2.3 mW, 514 nm laser. Three sets of devices were measured and the variation in the signal among them is about 10%. The signal observed corresponds to effective magnetic fields of 20, 16, -5, -18, 73, 36, -8 and -22 G for panels **a**,**b**,**c**,**d**,**e**,**f**,**g** and **h**, respectively. All plots are presented after subtracting a background signal, measured for the same devices but covered with the non-chiral 11-mercaptoundecylphosphoric acid (see Supplementary Fig. 8). Pink and light-blue areas correspond to ‘light on' and ‘light off' regimes, respectively.

The device was calibrated using an external magnetic field as shown in Supplementary Fig. 4. As expected from standard theory, no Hall voltage is observed in the absence of a magnetic field under dark conditions. However, as shown in Fig. 5, a Hall voltage appears - with either binding group, with two different sizes of NPs, and at both high and low temperature when the CdSe NPs are illuminated (with a 2.3 mW, 514 nm laser). Strikingly, the Hall voltage is of opposite sign at low and high temperatures.

## Discussion

**Figure 6:**
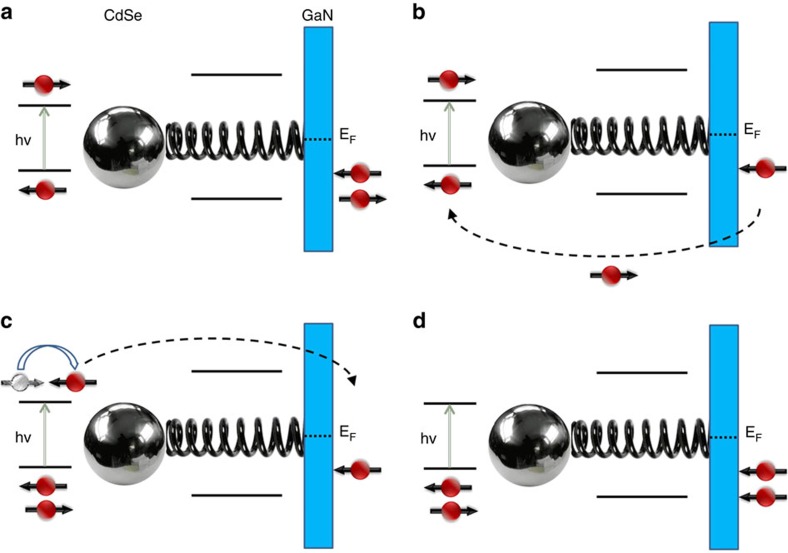
A schematic explanation of light-induced spin polarization at a semiconductor substrate adsorbed by nanoparticle (NP) bearing chiral molecules. (**a**) Photoexcitation of the NPs creates an electron–hole pair. (**b**) A spin-selected electron is transferred from the substrate to the hole on the NP. An unpaired electron is left on the substrate side. (**c**) Following spin randomization of the excited electron on the NP, this electron is transferred to the substrate. Both NP and substrate are again neutral. (**d**) A net spin polarization at the substrate is formed. This process may occur many times during the substrate spin-polarization lifetime, resulting in very high polarization. Depending on rate constants involved, step (**c**) may precede (**b**).

How can these results be explained and how are they related to the spin-polarization results of Fig. 1? A model explaining these results is given in Fig. 6. Upon excitation of the NPs (Fig. 6a), an electron is transferred from the substrate to the hole state on the NP (Fig. 6b). Because the oligopeptides are chiral, it is expected that the electron transfer process is spin selective, according to the above-explained CISS effect. An electron with the opposite spin is then accumulated on the device surface, acting to magnetize the substrate and create an effective magnetic field for the Hall measurement. If spin–orbit coupling in the NP is large (which is the case for CdSe), the spin of the photo-excited electron on the NP is randomized such that it is spin-selected for transfer to the substrate, still within the lifetime of the net spin formed on the substrate side (Fig. 6c). Both substrate and NP are now neutral again, but the net spin on the substrate is further enhanced (Fig. 6d). If the spin-lifetime in the substrate is long relative to that in the NPs (which is indeed the case for a GaN substrate and CdSe NPs), a specific spin is accumulated on the device surface, creating net magnetization^37^. A Hall voltage can then be measured. Its magnitude must be proportional to the spin accumulation field and its sign must reflect the type of spin accumulated. Under continuous illumination, the net effect will naturally depend critically on the lifetime of the spin state at the substrate surface, the number of spins being transferred between the substrate and the NP during this time, and the rate of spin-randomization. The device presented here is unique because it allows, in principle, a high spin-polarization without a net charge polarization. Therefore, high sensitivity is achieved here due to spins ‘loaded' onto the device without encountering the difficulty of Coulomb repulsions that one may face when trying to spin-polarize a medium by injecting spin-polarized electrons. In this sense, the situation is similar to the known case of spin–torque transfer used to spin-polarized ferromagnetic materials^38^.

With the model of Fig. 6 in mind, the results of Fig. 5 can now be rationalized. First and foremost, the spin transferred at room temperature is aligned parallel to the electron velocity, while at low temperature it is antiparallel to the velocity. This is in full agreement with an inversion of the CISS effect owing to the electrical dipole flip predicted theoretically in Fig. 3. Other insights can also be drawn from the figure. Clearly, the Hall signal is larger in the case of the phosphonate linker than in the case of the carboxylic one. This result is consistent with reports on the stronger binding of the phosphonates to GaAs^39^ and we assume, in light of these results, that the same applies to GaN. In addition, while at room temperature spin randomization is efficient both in the large and small NPs, due to phonon–spin interactions, at low temperature the signal is larger for small NPs. This can be explained by the quenching of the phonon–spin interaction and by the known faster spin-randomization in smaller NPs^40^,^41^, by as much as an order of magnitude, due to spin interaction with the surface^41^. Therefore, when the spin randomization in the NPs is fast, the NPs can be excited many times during the lifetime of the spin polarization on the GaN (see Fig. 5) and many spin-pairs can be injected into the GaN per NPs.

Using a calibration curve (see Supplementary Fig. 4), we can estimate the effective magnetic field that would be required for obtaining the same Hall voltage from an ordinary Hall measurement. The fields are found to vary between several Gauss to almost 100 G. Because the density of atoms in GaN is ∼1 × 10^23^ atoms per cm^3^ and the volume of the dielectric (40 μm × 700 μm × 22 nm) is ∼6 × 10^−10^ cm^3^, the total number of atoms in this volume is about 6 × 10^13^. A simple organization of 10^12^ spins can create such a magnetic field if they are equally spaced in a box of the size of the dielectric above the conduction channel. Because the number of spins in this volume is significantly smaller than the number of atoms (by more than an order of magnitude), we can safely assume that the exchange interaction between them, which may limit spin-polarization, is not important. This number of spins implies that ∼10^3^–10^4^ spins must be transferred from each adsorbed NPs during the lifetime of spin-polarization in the GaN, which is between tens to hundreds of nanoseconds^36^. The spin lifetime on the NPs is of the order of a few psec^40^,^41^. This ratio of lifetimes is about an order of magnitude more than that required to produce the above net spin polarization, showing that the system can indeed produce the required effective magnetic field by spin polarization that does not involve charge polarization.

**Figure 7:**
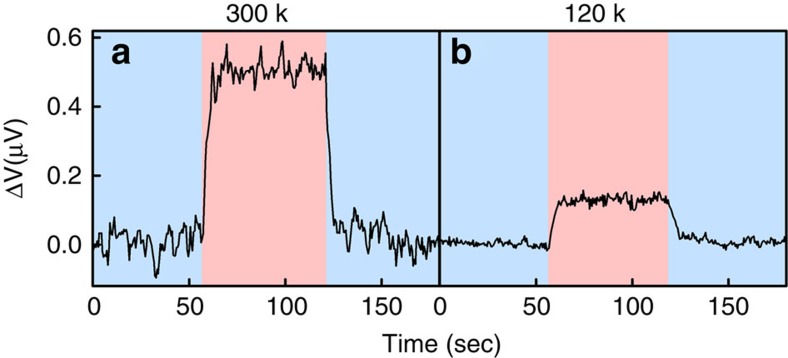
Hall potential measured for a mixed monolayer. Measurements were conducted at (**a**) 300 K and at (**b**) 120 K, at a current of 10 μA, for devices as in Figs 4 and 5, but with a mixed monolayer consisting of 40% NH_2_-{Ala-Aib}_8_-COCHNH_2_CH_2_PO_4_H_2_, 60% 11-mercaptoundecylphosphoric acid [HSCH_2_(CH_2_)_9_CH_2_PO_4_H_2_] molecules. The signal observed corresponds to effective magnetic fields of 11 and 3 G for the high and low temperature, respectively.

Last but not at all least: Beyond the computational evidence, can we establish a direct link between the above-discussed structural changes and the spin-transfer inversion? To probe this, the magnet-less Hall experiment was repeated with a *mixed* monolayer, consisting of 40% NH_2_-{Ala-Aib}_8_-COCHNH_2_CH_2_PO_4_H_2_ and 60% 11-mercaptoundecylphosphoric acid [HSCH_2_(CH_2_)_9_CH_2_PO_4_H_2_]. The phosphoric acid comprises alkane chains and therefore cannot form hydrogen bonds with the oligopeptide. Therefore the enthalpy gain expected from cold denaturation is much smaller and the effect should be much smaller, if not completely inhibited. The results are shown in Fig. 7. Indeed, for the mixed monolayers, the Hall voltage does not change sign upon cooling. Some signal reduction is observed at low temperature, which we attribute to the fact that the two molecules do not mix ideally and there may well be small domains that include the oligopeptides alone and exhibit denaturation as above. Still, the results of Fig. 7 lend very strong support to the theoretical prediction and close the circle between denaturation, dipole inversion, and spin-transfer inversion.

To summarize, using MD and DFT calculations we have been able to explain hitherto baffling experimental results that indicated inversion of the dipole and spin-filtering properties of chiral oligopeptide monolayers upon cooling. The calculations showed that the α-helix structure of the adsorbed oligopeptides ‘stretches' upon cooling, in a manner reminiscent of ‘cold denaturation' in biological systems, but where the monolayer plays the role of the solvent. Furthermore, this structural change induces an inversion of the peptide dipole, owing primarily to rearrangement of hydrogen bonds. Phenomenological theory of the CISS effect then explains that this dipole flip must be accompanied by a concomitant change in the spin that is preferred in electron transfer experiments. We have then supported the structural prediction via ellipsometry measurements and confirmed the link between the structural changes and the spin-filtering properties using a novel magnet-less, solid-state hybrid organic–inorganic Hall device, employed with oligopeptide monolayers based on alanine and aminoisobutyric acid. The results were further supported by control experiments on mixed monolayers. The results presented indicate that inter-molecular interactions may be of importance in defining the structure of adsorbed molecules within a monolayer and its temperature-dependence may be strongly related to monolayer packing. In addition, we introduced here a new device that can monitor structural changes in adsorbed chiral molecules by their spin filtering properties. In the future, this may serve to probe the role of spin in electron transfer through bio-systems and its dependence on system structure.

## Methods

### Device fabrication

A schematic representation of the AlGaN/GaN Hall devices and the setup is given in Fig. 4. The AlGaN/GaN Hall devices were fabricated by standard photolithography. The AlGaN/GaN HEMT Epi wafers on sapphire-substrates were purchased from NTT AT. The structure consist of the following layers, from the bottom up (see Fig. 4d): 1800, nm bulk i-GaN, 20 nm i-AlGaN, and a capping layer of 2 nm i-GaN. Ohmic contacts were achieved by e-beam evaporation of a standard Ti/Al/Ni/Au stack, followed by rapid thermal annealing at 900 °C. Device isolation was done by mesa etching using a BCl_3_/Cl_2_ base ICP-RIE (Plasma Therm).

### Formation of monolayers

Three organic monolayers were adsorbed as SAM on the GaN: 11-mercaptoundecylphosphoric acid (PCI Synthesis), NH_2_-{Ala-Aib}_8_-COCHNH_2_CH_2_PO_4_H_2_ and SHCH_2_CH_2_CO-{Ala-Aib}_5_-COOH (both from Genemed Synthesis Inc.). Solvents were reagent grade or better (Merck, Baker, or Bio-Lab). The GaN devices were sonicated prior to molecular adsorption in hot acetone and ethanol for 10 s each, then etched for 30 s in 6 M HCl, rinsed in water, and dried under a N_2_ stream. The samples were then cleaned and oxidized in UV/ozone oxidation for 30 min and placed in the adsorption solution (1 mM in toluene) immediately. Vials with the absorption solutions were filled with N_2_ and placed in a desiccator for 19 h in the case of 1-mercaptoundecylphosphoric acid and NH_2_-{Ala-Aib}_8_-COCHNH_2_CH_2_PO_4_H_2_, and for 65 h in the case of SHCH_2_CH_2_CO-{Ala-Aib}_5_-COOH. After adsorption, the samples were rinsed with toluene and dried with nitrogen.

In order to form a monolayer of CdSe NPs on the top of the SAM coated substrates, the devices were immersed for 4 h in a solution of anhydrous toluene (99.8%, Aldrich) containing core-only CdSe with a diameter of 6.2–7.7 nm (MK Nano) or 2.4–2.6 nm (NN-Labs). The samples were than sonicated for 5–10 s and washed with toluene to remove excess NPs. More details are provided in the Supplementary Methods.

### Monolayer characterization

**Figure 8:**
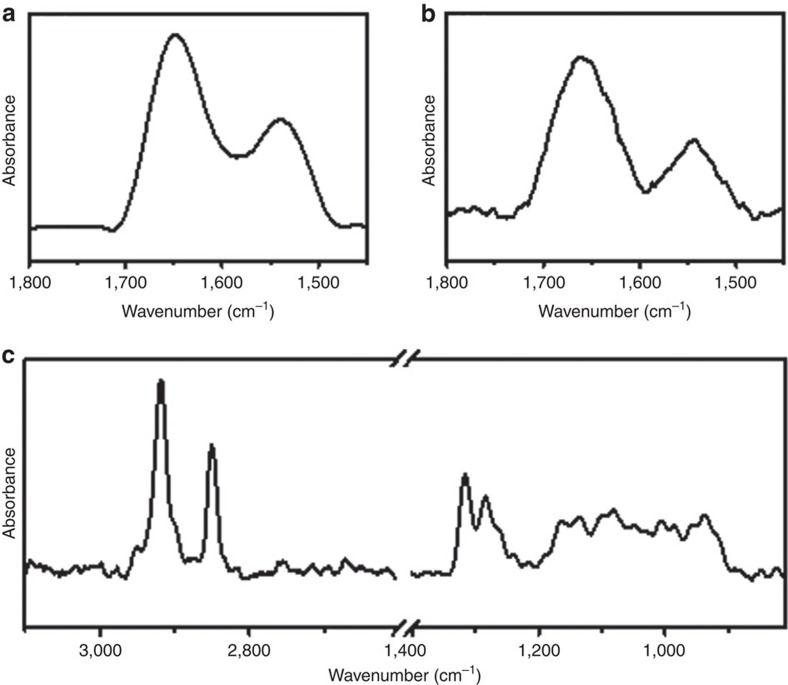
GATR-FTIR spectra of the adsorbed monolayers. (**a**) NH_2_-{Ala-Aib}_8_-COCHNH_2_CH_2_PO_4_H_2_, (**b**) SHCH_2_CH_2_CO-{Ala-Aib}_5_-COOH, and (**c**) 11-mercaptoundecylphosphoric acid monolayers adsorbed on gold.

Monolayer formation was confirmed by FTIR in grazing-angle attenuated total reflectance mode (GATR-FTIR), using a ThermoScientific FTIR instrument (Nicolet 6700) equipped with a VariGATR accessory (Harrick Scientific) and with a single reflection Ge crystal. The spectra of the two oligopeptides, SHCH_2_CH_2_CO-{Ala-Aib}_5_-COOH and NH_2_-{Ala-Aib}_8_-COCHNH_2_CH_2_PO_4_H_2_, exhibit the characteristic peaks that include stretching frequencies at 1659 and 1648, cm^−1^, related to the amide I band, and peaks at 1544 and 1539, cm^−1^ due to the amide II band (Fig. 8a,b). The spectrum of 1-mercaptoundecylphosphoric acid monolayer exhibits two large peaks at 2850 and 2917, cm^−1^, attributed to the symmetric and asymmetric CH_2_ stretching, respectively (Fig. 8c)^42^,^43^. A broad band between 1050 and 1200 cm^−1^ is related to the symmetric and antisymmetric P–O stretch and additional peaks associated with the P=O stretch appear at 1284 and 1315, cm^−1^ (ref. 44).

### Hall measurements

**Figure 9:**
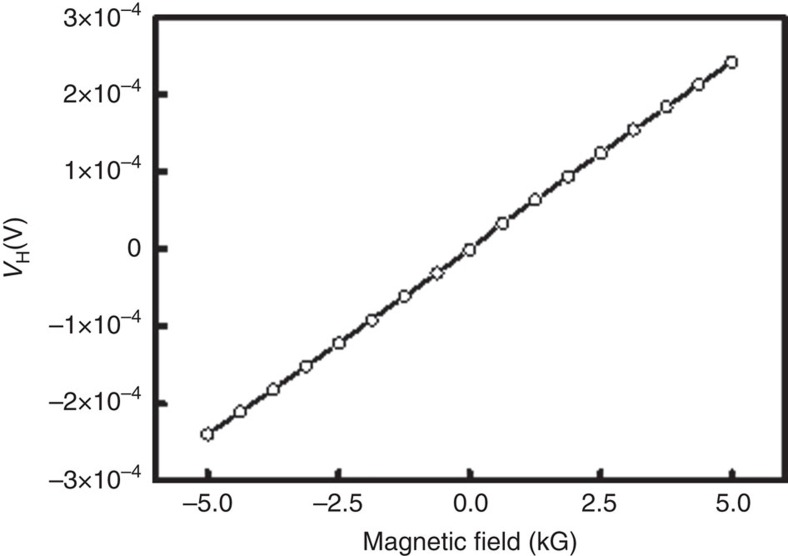
Hall voltage measured for calibrating the magnet-less Hall devices. The GaN-based device of Fig. 4 was measured, with the Hall voltage shown as a function of applied magnetic field, measured for a device coated with NH_2_-{Ala-Aib}_8_-COCHNH_2_CH_2_PO_4_H_2_ monolayer.

The GaN Hall device was attached to a sample holder and electrically connected to the measuring units. A constant DC current of 10 μA was applied along the device using a Keithley 6221 current source and the Hall voltage (*V*_H_) across the device was measured using a Keithley Nanovoltmeter 2182A (see Fig. 4). The resistance of the devices was in a range of 3–6 Ω. Chiral molecules were absorbed directly to the GaN, with CdSe NPs bound on top of them. The device was placed in between the magnetic poles and on a cold finger that could be cooled down to 14 K. A magnetic field of up to 0.5 T could be applied perpendicular to the sample plane by an electromagnet. The temperature of the sample holder was controlled by a temperature controller, with a temperature stability of 0.1% at 14 K and 0.3% at 300 K. For calibration, the conventional Hall voltage, as a function of external magnetic field, was measured for each device at several temperatures, 300, 120, 60 and 14 K. A representative plot taken at 300 K with a device coated with a monolayer of NH_2_-{Ala-Aib}_8_-COCHNH_2_CH_2_PO_4_H_2_ is shown in Fig. 9.

Several control experiments were performed with circularly polarized light on devices coated with the different monolayers and with large and small nanoparticles, as shown in Supplementary Figures 5,6 and on a bare device (see Supplementary Fig. 7) and with a device coated with 1-mercaptoundecylphosphoric acid and illuminated with circularly polarized light (see Supplementary Fig. 8).

### Molecular dynamics simulations of a polyalanine monolayer

A monolayer composed of 25 Ala10 polyalanines was prepared *in silico* by organizing the peptides initially in 5 × 5 arrays. The longitudinal axes of the peptides were oriented along the Z axis of the simulation box. The peptide molecules were neutralized at the N-terminal end (contained a protonated amino group) and at the C-terminal end (contained a protonated carboxyl group). To imitate the monolayer conditions, the N-terminal nitrogen of the amino group of each peptide was constrained in the Z dimension, which results in the ability of the peptides in the brush to move freely only in the XY plane. Any other peptide atoms were completely free to move. The peptides were attached in either an α-helical or a linear (extended β-strand) conformation.

The initial distance between neighboring peptides was chosen empirically, such that the neighboring chains of ALA10 were placed as closely as possible, but without inter-chain clashes. During the MD the peptides were free to move in the XY plane and change the distance between neighboring peptides. The designed monolayers were studied using all-atom molecular dynamics simulations using the GROMACS package, Version 4.5.4 (ref. 45) and with the CHARMM27 force field in vacuum conditions to mimic the dry environment of the monolayer^46^. In the initial step of the simulation conformations of the studied monolayers, the systems were relaxed using a conjugate gradient method of energy minimization. The LINCS algorithm^47^ was used to control bonds during the simulation. The Leapfrog algorithm was employed with a step of 2 fs.

The 5 × 5 monolayers were simulated for 10 ns (analyses of the trajectories showed that longer simulations did not result in additional conformational changes) with a time step of 2 fs (ref. 28). Temperature was controlled at 300 K using the Berendsen thermostat.

The conformations of Ala10 that were studied by DFT were generated by a pulling MD simulation when starting from a helical conformation. In this simulation, a force is applied at each terminal of the peptide at opposite directions. The pulling is done at rate of 0.01 nm/ps and with force constant of 1000, kJ per mol nm^2^. Several conformations were sampled along the transition from the α-helical to the β-strand structures, providing conformations with length varying between 15 and 32 Å.

### Density functional theory calculations of gas-phase oligopeptides

All gas-phase electronic-structure calculations were performed with Q-Chem^48^, version 4.0, using the correlation-consistent polarized valence triple zeta basis set. We used the optimally-tuned range-separated hybrid approach^15^, with a functional containing 80% short-range Perdew-Burke-Ernzerhof^49^ exchange, 20% short-range Fock exchange, 100% long-range Fock exchange and full Perdew-Burke-Ernzerhof correlation. The range-separation parameter was optimally tuned so as to obey the ionization potential theorem for the peptides in the neutral form in case of an unbound LUMO level, and to obey the same theorem in both neutral and anionic forms for a bound LUMO level.

## Additional information

**How to cite this article**: Eckshtain-Levi, M. *et al.* Cold denaturation induces inversion of dipole and spin transfer in chiral peptide monolayers. *Nat. Commun.* 7:10744 doi: 10.1038/ncomms10744 (2016).

## Supplementary Material

Supplementary Information Supplementary Figures 1-8 and Supplementary Methods

## Figures and Tables

**Table 1:** Monolayer thickness as a function of cooling time at 70K extracted from ellipsometry for a monolayer made from NH_2_-{Ala-Aib}_8_-COCHNH_2_CH_2_PO_4_H_2_ and adsorbed on Si/SiOx.

**Cooling time (min)**	**Thickness of molecule before cooling (Å)**	**Thickness of molecule after cooling (Å)**	**Difference in thickness (Å)**
30	16±1	20±1	4
60	18±1	25±1	7
120	16±1	29±1	13
