# Supplementary material for: Cold denaturation induces inversion of dipole and spin transfer in chiral peptide monolayers
Source: Nat Commun. 2016 Feb 26;7:10744. doi: 10.1038/ncomms10744 (PMC4773432; doi:10.1038/ncomms10744)
Supplement: Supplementary Information — Supplementary Figures 1-8 and Supplementary Methods [file ncomms10744-s1.pdf]

Supplementary Figures

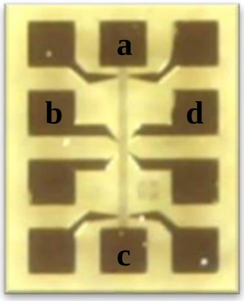

Supplementary Figure 1: Image of the GaN hall device.

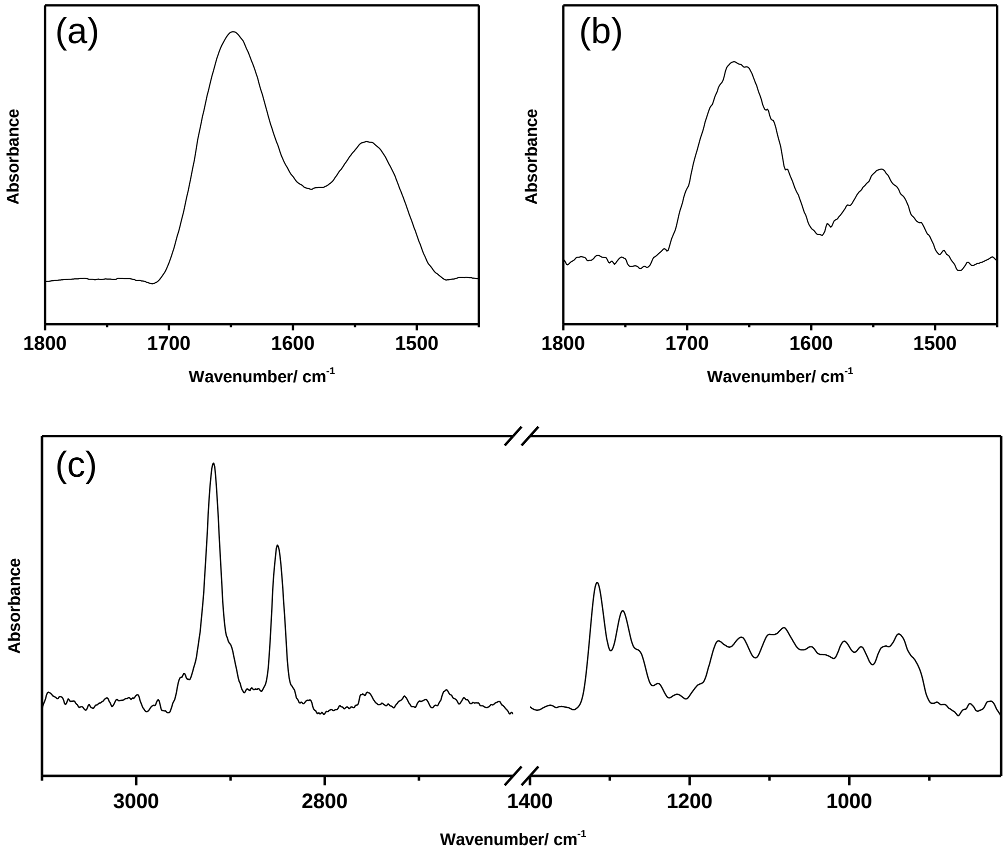

Supplementary Figure 2: GATR-FTIR spectra of NH2-{Ala-Aib}8-COCHNH2CH2PO4H2 (a), SHCH2CH2CO-{Ala-Aib}5-COOH (b) and 1-mercaptoundecylphosphoric acid (c) monolayres

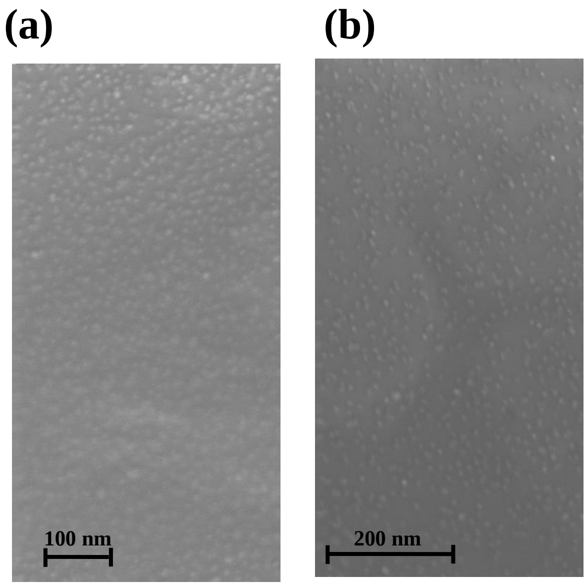

Supplementary Figure 3: SEM Images of the GaN substrate with CdSe NPs (7 nm) on top of SHCH2CH2CO-{Ala-Aib}5-COOH (a) and HSC11H24O3P (b) monolayers.

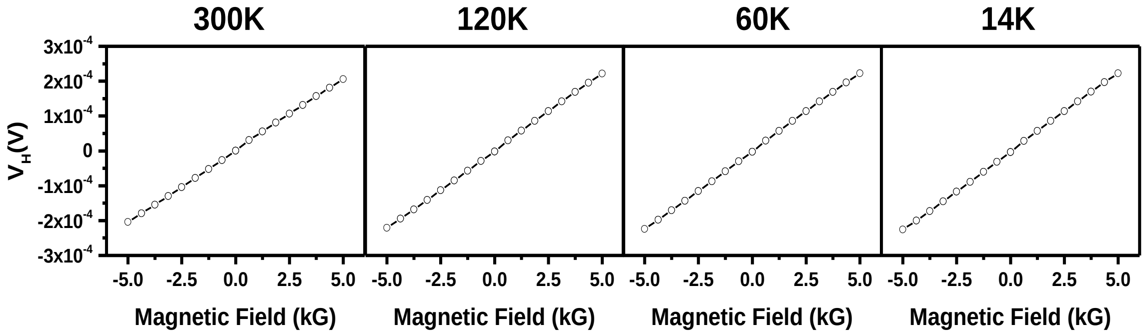

Supplementary Figure 4: V_H vs H curves of the hall device coated with monolayer of NH2-{Ala-Aib}8-COCHNH2CH2PO4H2 at diffrent temeprtures.

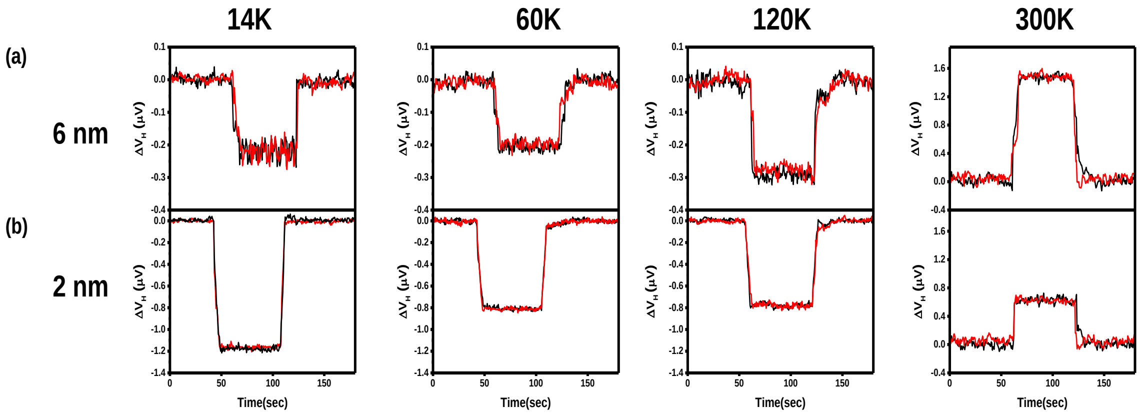

Supplementary Figure 5: Hall voltage as function of time measured on device with SHCH2CH2CO-{Ala-Aib}5-COOH during illumination with polorized light (left: red, right: black) at 514 nm, at diffrent tempertures; 14K, 60K, 120K, 300K, with two size of CdSe nanoparticles (a) 6nm and (b) 2nm at constant current of 10 µAmp.

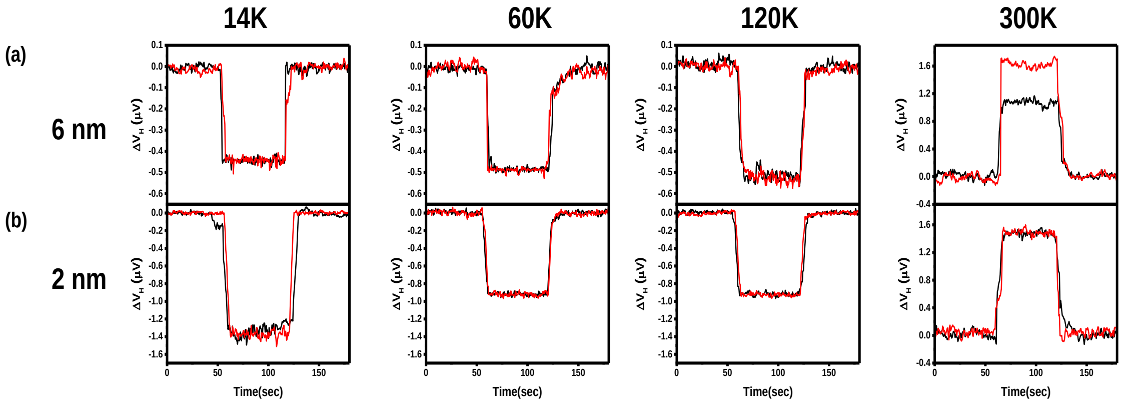

Supplement Figure 6: Hall voltage as function of time measured on device with NH2-{Ala-Aib}8-C3H6NO6P during illumination with polorized light (left: red, right: black) at 514 nm, at diffrent tempertures; 14K, 60K, 120K, 300K, with two size of CdSe nanoparticles (a) 6nm and (b) 2nm at constant current of 10 µAmp (After normilization with Hall voltage curves of 1-mercaptoundecylphosphoric acid hall device).

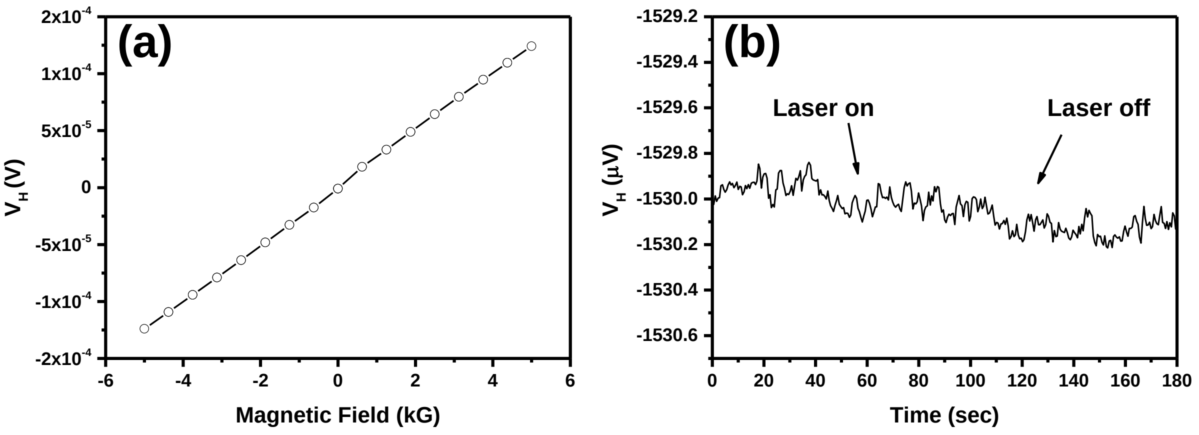

Supplementary Figure 7: (a) V_H vs H and (b) V_H vs time during illumination with green light at 514 nm measured on bare hall device at 300K with constant current of 10 μA .

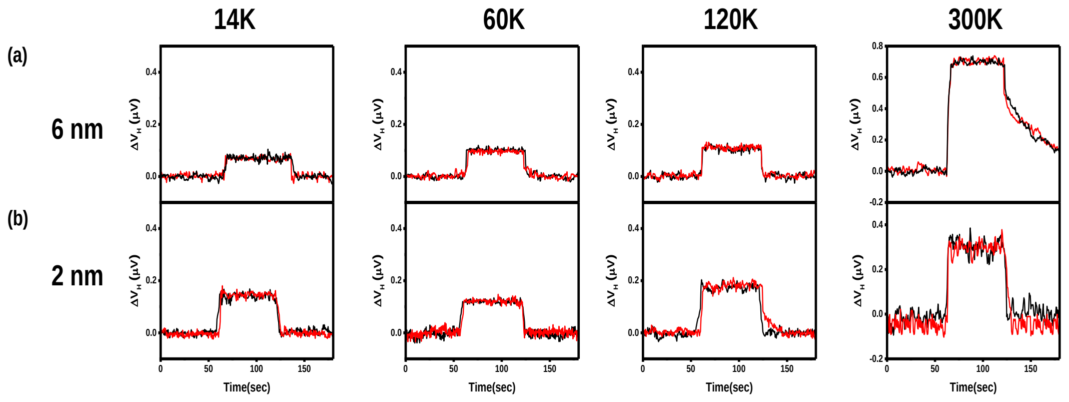

Supplementary Figure 8: V_H vs time measured on hall device with 1-mercaptoundecylphosphoric acid during illumination with polarized light (left:red, right: black) at 514 nm, at diffrent tempertures - 14K, 60K, 120K, 300K - with two size of CdSe nanoparticles: (a) 6 nm and (b) 2 nm at a constant current of 10 μA.

Supplementary Methods

Device Fabrication.

The AlGaN/GaN HEMT Epi wafer on sapphire-substrate was purchased from NTT AT. It consist of the following layers; first the nucleation layer, second i-GaN (1800 nm), third i-AlGaN (20nm), and a capping layer of i-GaN (2nm). The chip layout contains four devices. A schematic representation of the AlGaN/GaN Hall devices and the setup that was used in this work are given in Figure 3 and Supplementary Figure 1. The AlGaN/GaN hall devices were fabricated by photolithography in a class 1000 clean-room. Ohmic contacts were achieved by annealing at 900 C a standard Ti/Al/Ni/Au stack. Device isolation was done using a BCl_3/Cl_2 ICP-RIE. The width of the conducting channel is 40 μm, and the length is 700 μm. An ALD coating of 30 nm Al_xO_y was used as a gate dielectric and as a device passivation layer. An opening of this dielectic layer just above the sensing point was done by an etch solution of buffered HF (6:1) in the range of 18 to 24 μm^2.

Formation of monolayers.

Three Organic monolayers; 1-mercaptoundecylphosphoric acid, NH_2-{Ala-Aib}$_8$-$COCHNH_2CH_2PO_4H_2$ and $SHCH_2CH_2CO$-{Ala-Aib}$_5$-COOH were adsorbed on GaN according to the procedure detailed as follows. Solvents were reagent grade or better, purchased from Merck, Baker, or Bio-Lab. 1-mercaptoundecylphosphoric acid was purchased from PCI Synthesis, and NH_2-{Ala-Aib}$_8$-$COCHNH_2CH_2PO_4H_2$ and $SHCH_2CH_2CO$-{Ala-Aib}$_5$-COOH were purchased from Genemed Synthesis Inc. All chemicals were used without further purification. GaN devices were sonicated prior to molecular adsorption in hot acetone and ethanol for 10 s each, and then etched for 30 s in 6 M HCl, rinsed in water and then dried under a N_2 stream. After the samples were treated with UV/ozone oxidation (UVOCS) for 30 min, they were placed immediately in the adsorption solution (1 mM in toluene solution). The vials with the absorption solutions were filled with N_2 and placed in a desiccator for 19 h in the case of 1-mercaptoundecylphosphoric acid and NH_2-{Ala-Aib}$_8$-$COCHNH_2CH_2PO_4H_2$ while in the case of $SHCH_2CH_2CO$-{Ala-Aib}$_5$-COOH for 65 h. After adsorption, the samples were rinsed with toluene and dried with N_2 stream.

Nanoparticles adsorption.

In order to form a monolayer from CdSe NPs, the SAM coated substrates were immersed for 4 h in core-only CdSe with a diameter of 6.2-7.7 nm (MK Nano.) or 2.4-2.6 nm (NN-Labs) solutions in anhydrous toluene (99.8%, Aldrich). The samples were then sonicated (5 to 10 sec.) and washed with toluene to remove physiabsorbed NPs solution and dried with N_2 flow.

The fabrication of the monolayers was confirmed by surface characterization techniques such as; FT-IR spectroscopy, and SEM images of the NPs.

Fourier Transform Infrared (FT-IR) spectroscopy

Formation of the monolayers were confirmed by Fourier transform infrared spectroscopy (FTIR) in grazing-angle attenuated total reflectance mode (GATR-FTIR) using a ThermoScientific FTIR instrument (Nicolet 6700) equipped with a VariGATR accessory (Harrick Scientific) equipped with a single reflection Ge crystal. Spectra were collected by accumulating a minimum of 500 scans per sample with clean GaN surface as a reference and mounting the GaN sample at a Brewster angle of incidence of 67.4°. In addition, all spectra were collected while purging the VariGATR attachment and FTIR instrument with N_2 gas along the infrared beam path to minimize the peaks corresponding to atmospheric moisture and CO_2. Spectra were analyzed and processed using OMNIC software.

The spectra of the two of the oligopeptides $SHCH_2CH_2CO$-{Ala-Aib}$_5$-COOH and NH_2-{Ala-Aib}$_8$-$COCHNH_2CH_2PO_4H_2$ exhibit the characteristic peak a stretching frequency at 1659 and 1648 cm^{-1}, which is related to the amide I band, respectively, whereas the peak at 1544 and 1539 cm^{-1} is due to the amide II band (See Supplementary Figure 2a,b). In the aliphatic region, the spectrum of 1-mercaptoundecylphosphoric acid monolayer exhibit two large peaks at 2850 and 2917 cm^{-1} that are attributed to the symmetric and asymmetric CH_2 stretching vibrations, respectively (Supplementary Figure 2c). Furthermore, in the spectrum there is a broad band between 1050-1200cm^{-1} thus contributed to the symmetric and asymmetric mode of P-O stretch and a stretch mode of (P=O) at 1284 and 1315 cm^{-1}.

SEM images

A high-resolution scanning electron microscopic (SEM) SE2-detector images were produced with LEO-Supra-55VP. See Supplementary Figure 3.

Hall measurements

The GaN hall device was attached to a sample holder and electrically connected to the measuring units so that a constant current would passe through electrode a and c and hall voltage would be mesuered between b and d (Supplementary Figure 1). Chiral molelcules were absorbed throught the channel directly to the GaN and CdSe NPs were placed on top of them. The hall voltage were measured while illuminating light to the sample.

The device was placed in-between the magnetic poles and on a cold finger that could be cooled down to 14 K. A magnetic field up to 0.5 T could be applied perpendicular to the sample plane by an electromagnet. The temperature of the sample holder was controlled by a PID temperature controller with a temperature stability of 0.1% at 14 K and 0.3% at 300 K.

Typically, a dc current of 10 µAmp from a Keithley 6221 current source was passed through the device. The voltage drop across the junction was measured using a Keithley Nanovoltmeter 2182A device. For each devices the measured offset due to the misalignment between the Hall contacts lies below 6 Ω resistance.

Device characterization

The Hall voltage, V_H, as function of magnetic field, H, was collected for each device at several temp; 300, 120, 60, 14K. A representative plot for V_H vs H of hall device coated with monolayer of NH_2-{Ala-Aib}$_8$-$COCHNH_2CH_2PO_4H_2$ is shown in Supplementary Figure 4.

Supplementary Figure 5 shows hall voltage measured as function of time on device with $SHCH_2CH_2CO$-{Ala-Aib}$_5$-COOH during illumination with polorized light at 514 nm. It can be clearly seen that there is no diffrence in the two polorize light (left or right).

Supplementary Figure 6 Shows hall voltage measurements of NH_2-{Ala-Aib}$_8$-$COCHNH_2CH_2PO_4H_2$ hall device during illumination with polorized light at 514 nm. It can be clearly seen that there is diffrence in the two polorize light only at 300K and with large CdSe NPs (~6nm) (left or right).

Three control experimants were done; one with a bare device, and the second with a non chairl molecule 1-mercaptoundecylphosphoric acid (with two sizes of CdSe nanoparticles) (Supplementary Figures 7 and 8). Supplementary Figure 7 shows V_H vs H and V_H vs time during illumination with green light at 514 nm measured on bare hall device , at 300K and constant current of 10 µAmp.

Supplementary Figure 8 shows V_H vs time measured on hall device with 1-mercaptoundecylphosphoric acid during illumination with polorized light at 514 nm. It can be clearly seen that there is no diffrence in the two polorize light (left or right).
